# Lessons to be learned: best and worst results from a 7 years old clinical database of scoliosis patients

**DOI:** 10.1186/1748-7161-7-S1-O28

**Published:** 2012-01-27

**Authors:** S Negrini, S Donzelli, F Zaina

**Affiliations:** 1 ISICO, Milan, Italy

## Background and purpose

To verify which patients can reach the best and worst results during conservative treatment, since it is not yet known from previous researches [1,2].

## Material and methods

All scoliosis patients with more than 2 visits included in a prospective clinical database started in September 2003 were reviewed on August 31st 2010. A cut-off of 20 degrees (improvement or worsening) from the first observation was used to select patients. Patients were analysed for diagnosis, morphology, Cobb angle at start, curve improved/worsened, treatment, gender, Body Mass Index, clinical parameters.

## Results

Out of 1886 consecutive patients (TP), 62 (3.3%) changed 20° or more: 26 (1.4%) improved (range 20-34°) (IP), 36 (1.9%) progressed (20-60°) (PP).

Females prevailed in IP and low BMI in PP. In PP prevailed juveniles (35% vs 15%-23.8% in IP-TP); conversely, secondary scoliosis prevailed in both PP and IP (25%-15% respectively vs 1.9% in TP). In IP there were only patients who started over 30° Cobb (100%), while in PP 47% started between 10 and 19°; corresponding percentages in TP were 33.9% and 28.5% respectively. Diagnosis of thoracolumbar single curve was the most common in IP (46% vs 22.1% in TP), while double in PP (67% vs 49.8% in TP). Curves improved were thoracolumbar (IP: 58%), worsened thoracic (PP: 78%). Only patients who had a good or optimum treatment improved, but this was true also in 56% of progressed.

## Conclusions

Since these results are not similar to what would be expected according to the known natural history, conservative treatment appears able to change it.
